# Investigating the contribution of circulating inflammatory cytokines on the link between obesity and COVID-19

**DOI:** 10.1080/21623945.2025.2596403

**Published:** 2025-12-08

**Authors:** Zahra J. Khamis, Emmanouil Karteris, Amani Alhajeri, Steven G. Smith, Alexandra Blakemore, Fotios Drenos

**Affiliations:** a Department of Life Sciences, College of Health, Medicine and Life Sciences, Brunel University of London, London, UK; b Government Hospitals, The Genetics Center, Manama, Kingdom of Bahrain; c Department of Metabolism, Digestion and Reproduction, Imperial College London, London, UK; d College of Medicine, Nursing, and Health Science, University of Galway, Galway, Ireland

**Keywords:** Adipose inflammation, cytokines, adipokines, gene arrays and pathway analysis and chemokines

## Abstract

Coronavirus disease 2019 (COVID-19) is more severe in obesity. A cytokine storm was observed in critically ill patients. Since adipose tissue secretes cytokines, we investigated whether cytokines mediate the effect of obesity on COVID-19 severity. Using replicated two-sample Mendelian randomization analyses, we assessed the causal effect of body mass index (BMI) on COVID-19 severity. We evaluated the BMI effect on 41 inflammatory cytokines, JAK-2, lymphocyte percentage and leptin. We tested the relationship between these immunological factors and COVID-19 severity and conducted mediation analysis.Higher BMI increased the risk of COVID-19 severity. BMI was causally associated with five inflammatory cytokines – HGF, TRAIL, IL-13, IL-6, and IL-7 – with replication confirming these associations. TNF-α and IL-8 were identified as associated with COVID-19 severity, but with no replication support. Leptin-related genetic variation was associated with COVID-19 severity and supported by replication, but JAK-2 and lymphocyte percentage provided no evidence of association. None of the immunological factors tested showed consistent statistical evidence of mediation between BMI and COVID-19 severity. Our findings support the reported causal association between BMI and COVID-19 severity. Although several cytokines elevated due to higher BMI, we observed inconsistent evidence for baseline cytokines levels increasing COVID-19 severity. Baseline levels of circulating cytokines, JAK-2, lymphocyte percentage, and leptin showed no evidence of mediating the BMI and severe COVID-19 link. Limited participants in cytokine GWASs reduce statistical power, and missing population data on cytokine responses to infection are major limitations requiring resolution to explain cytokines’ mediating role between BMI and severe COVID-19.

## 1. Background

Obesity, the excessive accumulation of fat, is considered to be one of the major risk factors for developing severe COVID-19 [1]. Several studies indicate that, as body mass index (BMI) increases, so does the risk of mechanical ventilation and ICU admission [2]

Adipose tissue (AT), where body fat is stored, is an active immune organ that regulates hormonal, metabolic, and immune processes. AT plays an important role in the development of systemic and local low-grade inflammation that is recognized by local infiltration of immune cells, and by elevated circulating proinflammatory factors [3].

According to our current understanding, following infection with SARS-CoV2, resident macrophages are activated in adipose tissue. M1-polarized macrophage recruitment promotes the production of local and systematic pro-inflammatory cytokines (IL 8, IL 6, TNF α) resulting in a type-1 immune response (Th1) [4]. Individuals with obesity develop a chronic immune response in adipose tissue, resulting in the production of B cells, T cells and natural killer (NK) cells that can produce a cytokine storm. A cytokine storm is the result of the activity of several immune cells including dendritic cells, macrophages, natural killer cells and T and B lymphocytes. Upon viral infection, the innate immune cells recognize the molecular structure of the invading virus, called pathogen associated molecular patterns (PAMPs), by pattern recognition receptors (PRRs) around the cells. The interaction between PRRs and PAMPs triggers signalling pathways that induce the expression of genes encoding proinflammatory cytokines [5]. The sudden increase in cytokines, ‘the cytokine storm’, results in accumulation of immune cells into the infection site, causing tissue damage. This can include damage to endothelial cells, the vascular barrier, and its capillaries, as well as diffuse alveolar damage, which may lead to multiorgan failure, and death [6].

The immune response cascade is also affected by other key players such as: leptin; Janus kinase 2 (JAK2); and lymphocytes [7]. As well as regulating appetite and reproductive functions, leptin is a proinflammatory adipokine secreted by adipose tissue. Thus, leptin contributes to inflammation and may lead to chronic inflammation [7]. It directly stimulates macrophages (M1 phenotype), natural killer (NK) cells, dendritic cells (DC) and T-helper cells to secrete proinflammatory cytokines such as IL1, IL6, and TNFα [8].

The Janus kinase (JAK)/signal transducer and activator of transcription (STAT) pathway is the most important signalling pathway of the leptin receptor. Upon leptin binding, JAK2 activation occurs, which leads to STAT3 binding to phosphorylated tyrosine in leptin receptor followed by expression of responsive genes [9]. JAK2 might be imbalanced in patients with obesity could lead to an exaggerated immune response [9].

Lymphocytes, mainly CD4+ T cells and CD8+ T-cells, play an essential role as antiviral immunity [10]. Patients who had recovered from COVID-19 had high to normal levels of T-cells [11]. In severe cases of SARS-CoV2, patients have low levels of lymphocytes, mainly CD8+ [10]. Therefore, lymphocyte counts have been suggested as possible biomarkers to evaluate the severity of COVID-19 in patients [11].

The link between obesity and COVID-19 severity has been hypothesized as being, at least partly, due to the immune responses of visceral fat in patients with obesity [6]. There have been several reports describing the effects of inflammatory cytokines on COVID-19 infection [12] and the role of obesity in increasing the severity of COVID-19 [10,11].

To test the mediating role of cytokines and related immunological markers in the relationship between BMI and COVD-19 severity, we need to first understand the causal relevance and direction of effects between the different factors. Mendelian randomization (MR) allows us to use genetic variants associated with an exposure of interest as instrumental variables to assess causality. When used as a two-sample approach, summary results from large genome-wide association studies (GWAS) can be used, significantly increasing the number of people we can include in the analysis and, thus, improving our statistical power to detect a modest effect on an outcome [13].

In this study, using publicly available data from exceptionally large GWAS including hundreds of thousands of individuals and the Mendelian randomization methodology, we aim to investigate the potential mediation role of 41 cytokines, leptin, JAK2, and lymphocyte percentage in the link between obesity and COVID-19 severity. Identifying the mediators of the effect of obesity on COVID-19 severity will allow us to provide epidemiological evidence for targeting specific cytokines that might decrease the risk of COVID-19 on patients with obesity, and which might also have wider consequences for other categories of patients.

## 2. Materials and methods

### 2.1. Study design

**Figure 1.**
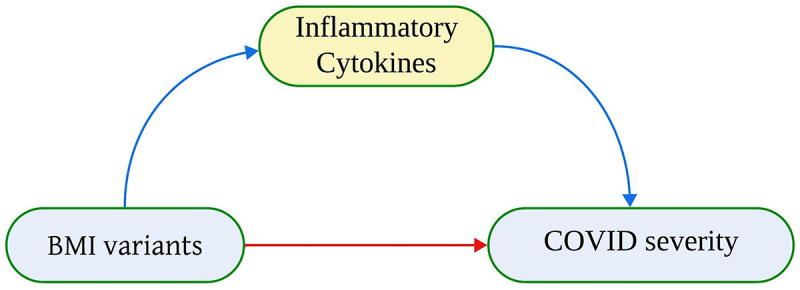
A simplified illustration of total effect, direct, and indirect effect using two sample Mendelian randomization. Direct effect between exposure (BMI variants) and outcome (COVID-19 severity) is C’. Indirect effect between exposure and outcome through mediator (cytokines) is a’+b’. The total effect is direct effect + indirect effect.

The study was performed through three MR analyses as outlined in Figure 1 MR analysis was used to estimate the causal association between BMI and severe COVID-19 [2]. We also used MR analysis to test the effect of BMI on circulating cytokines and related traits [3]. We conducted MR analysis to identify the circulating cytokines and related traits causally relevant to COVID-19 severity. Finally, we tested whether any of the factors identified as associated with BMI and COVID-19 severity shows evidence of a mediating role between the two, using a multivariable MR and estimating the possible mediating effect.

To ensure the accuracy of our outcomes and to reduce false positives, replication analyses were performed for the statistically significant results identified.

### 2.2. Selection of genetic instrument variables for BMI

The genetic instruments for BMI were extracted from the Metabochip meta-analysis and genome-wide association study conducted by Locke et al. The study identified 289 BMI-associated loci (at a p-value threshold of <5 × 10 ^−8^) from 322,154 participants of European descent [14].

### 2.3. GWAS summary statistics for COVID-19 severity

COVID-19 severity genetic association data were obtained from the COVID-19 host genetics initiative (COVID-19 HG) GWAS meta-analysis [15] (https://www.COVID-19hg.org/). The COVID-19 HG team has made summary data publicly available on multiple COVID-19 outcomes from several studies. The data acquired for this analysis is with an accession ID: ‘GCST011073’ at the GWAS Catalog of European populations. The study is from 13 May 2020, which is the last time point available before the wide release of the COVID-19 vaccination. Severity of infection is defined as hospitalized COVID-19 vs. not hospitalized COVID-19 release 5 and includes 38,984 European ancestry cases and 1,644,784 European ancestry controls. The relevant data can be downloaded from (https://www.ebi.ac.uk/gwas/studies/GCST011073) [15].

### 2.4. GWAS summary statistics for inflammatory cytokines and C-reactive protein

The summary statistics for inflammatory cytokines were acquired from the work of Kalaoja et al. [12]. The authors analysed a cytokine panel (41 cytokines) from the National FINRISK Study (1997 and 2002 cohorts) and the Cardiovascular Risk in Young Finns Study (YFS). The full GWAS summary results for all 41 inflammatory cytokines are available through the University of Bristol (https://data.bris.ac.uk/data/dataset/3g3i5smgghp0s2uvm1doflkx9x) [16].

### 2.5. GWAS summary statistics of inflammatory cytokines used for replication analysis

deCODE Genetics cohort: the genomic and statistical data of 39 inflammatory cytokines were obtained from 35,559 Icelanders included in deCODE [17]. SOMAScan assay was carried out to measure cytokines levels in plasma samples. Proteomic raw data were downloaded from https://www.decode.com/summarydata/.

### 2.6. GWAS summary statistics for leptin levels, lymphocytes percentage and tyrosine-protein kinase JAK2 levels

The summary statistics for lymphocyte percentage of leukocytes were obtained from the study by Jung H. et. al. [18] based on data of 174,488 individuals from the UK Biobank with European ancestry (U.K.). Lymphocyte percentage was measured by dividing the number of lymphocytes in blood by the total number of white blood cells.

Summary statistics data for leptin levels were acquired from the published study by Yaghootkar H. et al. [19] accession ID: ‘GCST90007310’ and downloaded from the GWAS Catalog https://www.ebi.ac.uk/gwas/studies/GCST90007310. Yaghootkar et al. analysed circulating leptin levels in 49,909 European ancestry individuals for association with exonic genetic variants [19].

GWAS of tyrosine-protein kinase (JAK2) levels was obtained from a genome-wide association study of serum proteins published by Gudjonsson et al. [20], accession ID: ‘GCST90086866’ and was downloaded from the GWAS Catalog https://www.ebi.ac.uk/gwas/studies/GCST90086866. The authors performed a GWAS for 5,355 European Icelandic ancestry individuals with 2,091 serum proteins including JAK2 measured through the SOMAmer platform [20].

### 2.7. GWAS summary statistics used for replication analysis of leptin and JAK2 levels

*Leptin GWAS:* Summary statistics data for leptin levels were taken from the published study by Folkersen L. *et al*. [21] accession ID: ‘GCST90012076’ and downloaded from the GWAS Catalog https://www.ebi.ac.uk/gwas/studies/GCST90012076.Folkersen analyzed circulating levels in 21,758 European ancestry individuals using Genome-wide genotyping array.

### 2.8. Statistical analysis

All analyses were performed using R [22]. The TwoSampleMR package [23] was used for MR and the ggplot2 package [24] for plots, unless otherwise stated. The inverse variance weighted (IVW) approach was considered as the main MR analysis for estimating the causal relationships unless there was evidence for directional pleiotropy. Directional pleiotropy was tested through the intercept term of the MR Egger analysis method [25]. In the presence of pleiotropy, we used the weighted median estimate. All p-values with *p* < 0.05 were considered statistically significant. All association estimates were reported as either odd ratios (OR) with 95% confidence intervals (CIs) for binary outcomes or as beta coefficients and 95% CIs for continuous outcome variables. The mediation analysis was performed as described in the supplementary material of Burgess et al. [26]. Causal Mediation analysis is a method that assesses a hypothetical causal effect of exposure and outcome via a mediator. It separates the total effect into direct and indirect effect whereby the indirect effect includes the hypothetical mediator to the outcome. The total effect is obtained from the two main sample MR results. The direct effect of BMI on COVID-19 severity was estimated using a Multivariable Mendelian randomization using the MVMR package in R. Multivariable MR analysis allows the simultaneous estimation of causal effects of multiple exposures on an outcome [27]. The indirect effect can then be estimated as the difference between the total and direct effects (Figure 4).

To measure the strength of genetic instruments, we approximated the F-statistic for each selected independent SNPs in inflammatory cytokines using the squared ratio of the effect size (β) over the standard error from GWAS summary data.

### 2.9. Colocalization analysis

After MR analysis, we conducted colocalization analysis to determine whether the same genetic variants cause the resulted observed associations between inflammatory cytokines levels and COVID-19 severity. Colocalization is an important sensitivity analysis step to exclude linkage disequilibrium (LD) confounding in a locus.

Colocalization was performed using the coloc R package (version: 5.2.3) which estimates the posterior probability that two related traits share the same causal variant in a genomic area. Here, we followed the enumeration colocalization approach using the Bayesian framework to test five alternative hypothesis of H0: no association in the area, H1: only trait 1 (exposure) is associated with the genomic area, H2: only trait 2 (outcome) has an association in the area, H3: both trait 1 and 2 are associated with the area but with different causal variants, and H4: both traits are associated with the same causal variant [28]. In an MR setting, when the instrument is chosen to be associated with the exposure but with no consideration of its association with the outcome, H1 can be considered the most likely hypothesis when statistical power in the outcome GWAS is limited. Even under this scenario the ‘balance of probability’ between H3 and H4 can help us understand if the chosen instrument is likely violating the MR assumptions due to LD confounding. This is more important in cases of a statistically significant result, so we applied colocalization analysis to the inflammatory cytokines that showed significant causal associations with COVID-19 severity. For each cytokine, we run single locus colocalization using GWAS data of both the corresponding cytokine and COVID-19 severity (release 5, study ID ebi-a-GCST011073) [29]. A total of 150 loci were analysed for TNF α and 149 loci for IL 8, with 114 loci and 125 loci retained after harmonization with the outcome results. The independent loci for both cytokines were identified via clumping from 1000 Genomes project on European reference panel [30]. The colocalization probabilities for H3 and H4 were compared per SNP and their difference tested using a Wilcoxon paired test, a non-parametric alternative of the t-test.

## 3. Results

### 3.1. The causal effect of BMI on COVID-19 severity

From the previously reported 289 BMI associated SNPs, 187 remained after removing variants with LD of r^2^ > 0.001 with any other SNP. All 187 were found in the COVID-19 severity data and were successfully harmonized between the two sets of results. The inverse variance weighted (IVW) estimates showed that BMI was positively associated with COVID-19 hospitalization (OR = 1.242; 95% CI: 1.034–1.317, *p* = 1.63 × 10^−8^) Figure 1. MR Egger was used to test the existence of pleiotropy, and no evidence of directional pleiotropy was present (Intercept 0.0005, *p*-value = 0.638).

### 3.2. The effect of BMI on inflammatory cytokines

**Figure 2.**
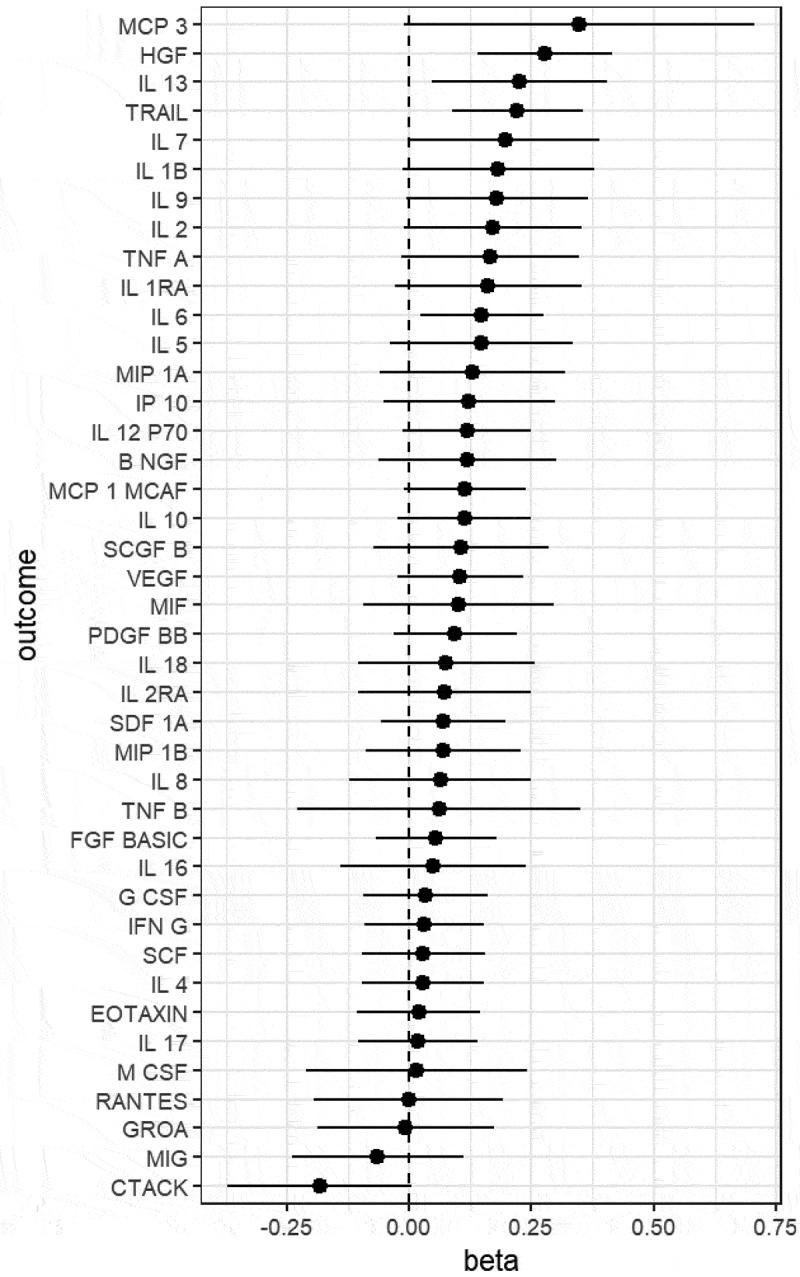
Causal effect (betas) from the two sample Mendelian randomization analysis of body mass index (BMI) (exposure) with inflammatory cytokines (outcome).

We estimated the effect of BMI on 41 inflammatory cytokines. Genetically predicted BMI was positively associated with five inflammatory cytokines with P-value less than 0.05 as shown in Supplementary Table S1 and Figure 2. The cytokine with the highest magnitude of effect from BMI was hepatocyte growth factor (HGF) (β = 0.277, se = 0.0703, *p* = 8.00 × 10^−5^). Other cytokines affected included TNF-related apoptosis-inducing ligand (TRAIL) (β = 0.221, se = 0.0678, *p =* 1.09 × 10^−3^), IL 13 (β = 0.226, se = 0.0916, *p =* 1.38 × 10^−2^), IL 6 (β = 0.148, se = 0.0638, *p =* 2.01 × 10^−2^) and IL 7 (β = 0.196, se = 0.0992, *p =* 4.83 × 10^−2^). None of the cytokines tested showed evidence of decreasing with increasing BMI as shown in Figure 2 and Supplementary Table S1, though CTACK was close (b = −0.184, se = 0.097, *p* = 5.6 × 10^−2^). The replication Mendelian randomization results using deCODE confirmed the identified associations: HGF (β = 0.208, se = 0.034, *p =* 8.31 × 10^−10^), TRAIL (β = 0.115, se = 0.0298, *p =* 1.22 × 10^−4^), IL 13 (β = 0.135, se = 0.0304, *p =* 9.27 × 10^−6^), IL 6 (β = 0.133, se = 0.0293, *p =* 5.90 × 10^−6^), and IL 7 (β = 0.0856, se = 0.0297, *p =* 3.99 × 10^−3^).

### 3.3. The effect of inflammatory cytokines on COVID-19 severity

**Figure 3.**
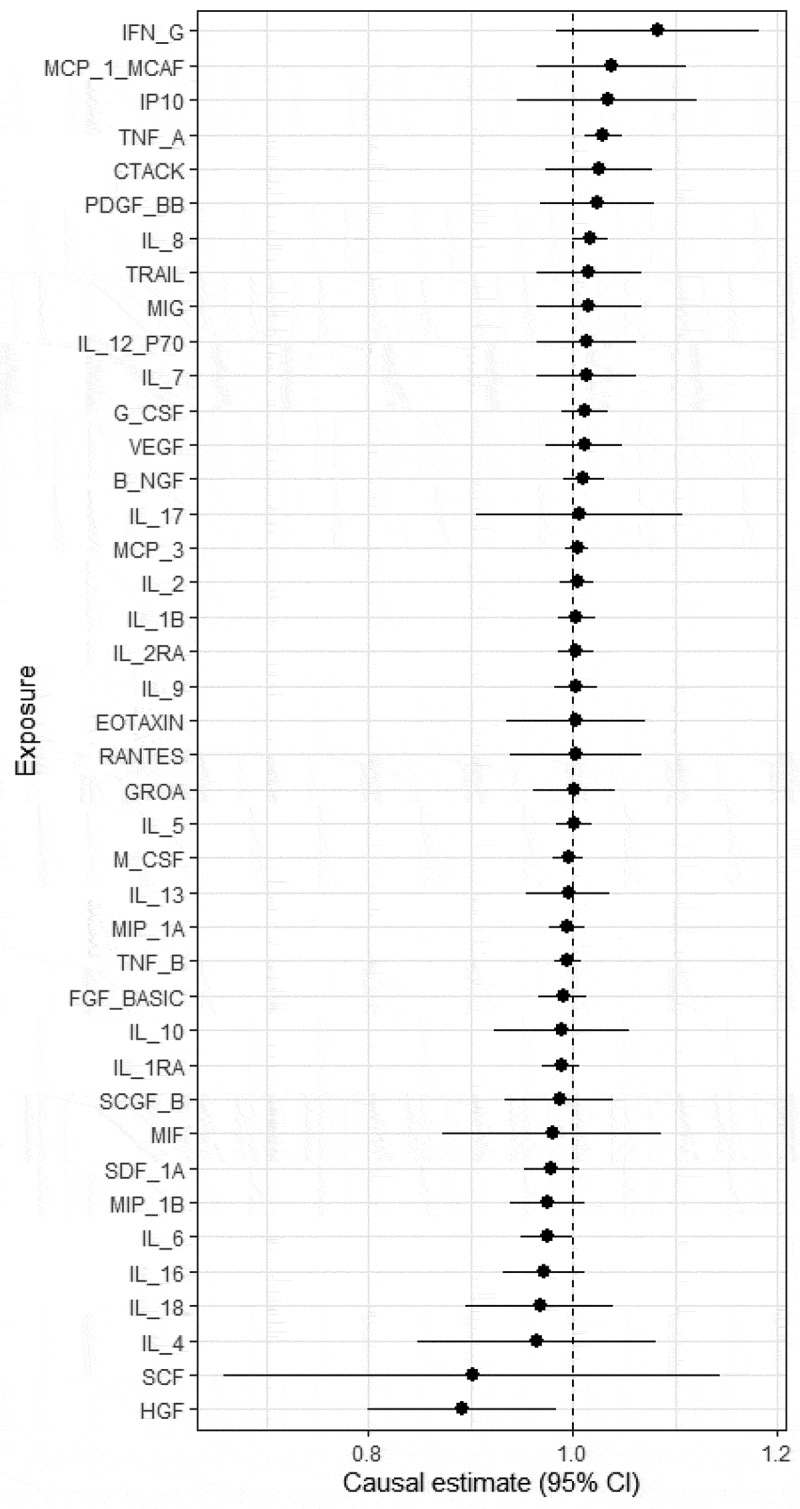
Causal effect estimates (odds ratios and 95% confidence intervals) from the two-sample Mendelian randomization analysis of inflammatory cytokines (exposure) with COVID-19 severity (outcome).

We tested all 41 inflammatory cytokines for a causal association with COVID-19 severity. Of these, two cytokines were positively associated with severity of COVID-19: TNF α (OR = 1.030; 95% CI: 1.011–1.048, *p* = 1.72 × 10^−3^) and IL 8 (OR = 1.017; 95% CI: 1.000–1.034, *p =* 4.88 × 10^−2^). Neither associations were replicated: TNF α (OR = 0.894; 95% CI: 0.610–1.774, *p* = 0.438) and IL 8 (OR = 0.980; 95% CI: 0.861–1.099, *p =* 0.747). On the other hand, HGF (OR = 0.892; 95% CI 0.800–0.984, *p =* 1.46 × 10^−2^) was the only cytokine with evidence of being inversely associated with COVID-19 severity as shown in Figure 3 and Supplementary Table 2. HGF replication did not show evidence of inverse association with COVID-19 severity (OR = 0.992; 95% CI 0.875–1.105, *p =* 0.869). We found no evidence of directional pleiotropy for any of the associations tested using the intercept of the MR Egger model (Figure 3 and Supplementary Table 2). We evaluated the instrument strength of by calculating the F-statistics of harmonized SNPs of around 18 inflammatory cytokines. The F-statistics range is approximately 300 across cytokines and the average of 12.2 which is above the F-statistics conventional threshold of 10. This indicates that weak instrument bias is not affecting the results.

Colocalization of individual genomic blocks of SNPs associated with our instruments revealed that H1, the genetic variant is associated with only the exposure, was the most likely outcome (Supplementary Table 5 & 6). Between H3 and H4, the exposure and outcome associated with different or the same genetic variant alternatively, H3 was statistically significant more likely than H4.

### 3.4. The effect of BMI on each of leptin, JAK2 and lymphocyte percentage

We analysed the effect of BMI variants individually on leptin, JAK2, and lymphocyte percentage. Genetically predicted BMI was only statistically significantly associated with leptin (β = 0.732, se = 0.0798, *p =* 4.756 × 10^−20^). JAK2 (β = 0.142, se = 0.101, *p =* 0.16) and lymphocytes (β = 0.0475, se = 0.0308, *p = 0*.12) showed no evidence of causal associations. The observed causal association with leptin was confirmed by our replication: leptin (β = 0.562, se = 0.0405, *p =* 8.66 ×10^−44^).

### 3.5. The effect of leptin, JAK2 and lymphocyte percentage on COVID-19 severity

Leptin-associated genetic variants were positively associated with COVID-19 severity (OR = 1.289; 95% CI: 1.0766–1.501, *p =* 1.92 × 10^−2^), whilst no statistically significant evidence was found between SNPs for JAK2 (OR = 1.006; 95% CI: 0.950–1.061, *p* = 0.83) and lymphocyte percentage (OR = 0.989; 95% CI: 0.908–1.069, *p* = 0.78) with COVID-19 severity. Although just above the p-value threshold and given the very close replicated estimate, we consider the association between leptin and COVID-19 severity as confirmed (OR = 1.222; 95% CI: 1.021−1.424, *p =* 5.08 × 10^−2^).

### 3.6. Mediation analysis

**Figure 4.**
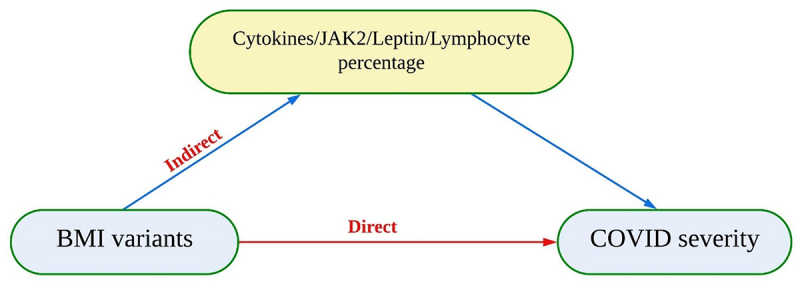
A simplified illustration of mediation analysis, direct, and indirect effect using two sample Mendelian randomization. Direct effect between exposure (BMI variants) and outcome (COVID-19 severity). Indirect effect between exposure and outcome through mediator (cytokines, JAK2, leptin and lymphocytes percentage).

Since no single cytokine measure was associated with both BMI and COVID-19 severity, we measured the indirect causal effect of BMI on COVID-19 severity via all inflammatory cytokines together. Leptin was associated with both BMI and COVID-19 severity, and we also tested its mediation effect (Figure 4).

### 3.7. MVMR of BMI and all inflammatory cytokines on COVID-19 severity

To examine the possible mediation effect of all inflammatory cytokines together, and due to the strong correlation between cytokines, we followed a stepwise approach in building our MVMR model by adding inflammatory cytokines, guided by the knowledge of immune response pathways to identify those contributing independent information and exclude those causing collinearity. Four non-correlated cytokines were included in the final model, BNGF, GROA, IL2RA, and MIP1A (Supplementary Table 3). The direct effect of BMI (103 SNPs) on COVID-19 severity was no longer statistically significant (OR = 0.969; 95% CI: 0.812–1.125) (Supplementary Table 3), but none of the inflammatory cytokines was statistically significant with COVID-19 severity (Supplementary Table 3).

### 3.8. MVMR of BMI and leptin levels on COVID-19 severity

When leptin was included as a conditional factor in the BMI to COVID-19 severity MR model through MVMR, the direct effect of BMI (26 SNPs) on COVID-19 severity was no longer statistically significant (OR = 0.9839; 95% CI: 0.752–1.216).

### 3.9. Causal mediation analysis

The mediation of the joint inflammatory cytokines was tested. The BMI effect mediated by the cytokines included in the model was estimated as 0.102 (CI: −0.0542, 0.2585), but the confidence intervals include 0, indicating a lack of statistical significance for the estimate (Supplementary Table 4). This represents 52.8% of the total BMI effect estimated, but despite its large value we were not able to find statistical evidence of a mediation effect.

Mediation analysis was performed for leptin as it showed evidence of positive association with both BMI and COVID-19. We re-estimated the total effect using the BMI SNPs from the MVMR and leptin causal mediation analysis also showed no evidence of a significant association, indirect effect of 0.129 (CI: −0.1592, 0.4172). Again, although this was 19.7% of the BMI effect on COVID-19 severity, we lack the statistical evidence to confirm its presence (Supplementary Table 3).

## 4. Discussion

Several studies have identified obesity, particularly as measured by high body mass index (BMI), as a risk factor for COVID-19 severity. Since obesity also promotes inflammation, the contribution of cytokines in increasing the risk of severe COVID-19 has been hypothesized, but is not yet well understood.

Severe forms of COVID-19 are also associated with an overreaction of the immune system through a cytokine storm. Here, using a two sample Mendelian randomization approach, we confirmed the causal link between obesity, as measured by high BMI, with COVID-19 severity in the form of hospitalization following an infection. We also established the causal effects of genetically predicted BMI on cytokines and of cytokines to COVID-19 severity. We investigated the effect of BMI on leptin, JAK2 and lymphocyte percentage and their effect on COVID-19 severity. Mediation analysis was performed for the examined risk factors on the link between BMI and COVID-19 severity and although a large proportion of the link between the two can potentially be explained by a combination of cytokines and leptin, we were not able to find clear statistical evidence to support their mediating role.

Our results agree with both observational and MR studies done previously between BMI and COVID-19 severity: persons with obesity are at higher risk of severe course of COVID infection. The effect we estimated (OR = 1.207; 95% CI: 1.115–1.307) is close to that reported by several Mendelian randomization studies of BMI and COVID-19 severity. COVID-19 severity in high BMI patients was also observed in a previously published meta-analysis of 167 studies including 3 million COVID-19 patients. The meta-analysis was performed to compare the association between high BMI hospitalized patients and normal BMI COVID-19 hospitalized patients and the outcome results showed that severity of COVID-19 is 1.5 times higher in obese patients compared to non-obese patients. The slight overestimation of the effect of BMI indicates the possible presence of confounding factors between the two. Further support for the causal relationship of obesity to COVID-19 severity was seen in studies showing a decrease in COVID-19 severity in patients following bariatric surgery [31].

We assessed 41 inflammatory cytokines to investigate possible mediators that may drive a more severe reaction to the SARs-COV-2 in individuals with obesity. We found five inflammatory cytokines affected by elevated BMI levels (HGF, TRAIL, IL 13, IL 6, and IL 7). These were able to validate further in another independent data set. Supporting our finding, the link between obesity and changes in the cytokine profile has also been previously reported [32]. Further, Dam et al. reviewed an increase of proinflammatory cytokines; IL 1, IL 6, and TNF α by inducing immune cell infiltration from adipose tissue in cell culture settings using human primary adipocytes and found adipose cells are capable of promoting an inflammatory state [33]. A two sample Mendelian randomization performed by Kalaoja et al. identified HGF, MCP-1, and TRAIL as significantly associated with BMI variation [12]. The increase of the affected cytokines seen in our study is probably due to larger number of independently significant BMI variants used compared to other studies.

We carried out two sample Mendelian randomization of inflammatory cytokines with COVID-19 severity and our results indicated causal associations with TNF α and IL 8. Both inflammatory cytokines were investigated in several studies reporting the production of high levels of several inflammatory cytokines as part of initiation of cytokine storm in COVID-19 infection such as IL 2, IL 6, IL 7, IL 8, IL 10, GSCF, IP10, MCP 1, MIP1 A and TNF α [34–36]. Although both IL 8 and TNF α have been previously reported as associated with COVID-19 and identified by our primary MR as increasing the risk of more severe COVID-19, our replication analysis fails to further support their causal role. Our colocalization analysis also showed that these results may have been affected by LD confounding which may explain the observed inconsistency between the discovery and replication results if LD pattern differences exist between the independent samples used. This is possible due to the unique genetic patterns of Icelandic populations used in the replication [37]. Both studies used European populations, Icelandic Cohort has larger sample size (35,559 vs ~8,293 individuals in Finnish Cohort). Although both used Illumina for genotyping arrays, different measurement platforms were applied. FINRISK cohort used traditional immunoassays while Icelandic cohort employed the SOMAscan assay. These assay differences may affect results comparison. Furthermore, FINRISK cohort used haplotype reference consortium (HRC) panel in imputation, while they used whole genome sequencing of Icelanders as population reference panel [12,17].

Our findings suggest that further analysis of cytokines in more harmonized and larger multi-ancestry GWAS datasets, as currently available for BMI and COVID-19, is required.

We also carried out mediation analysis to investigate the role of inflammatory cytokines on the link between BMI and COVID-19 severity. We found no statistical evidence of mediation by inflammatory cytokines despite the potentially very large, estimated proportion of the mediated effect. Although several studies suggested a role for inflammatory cytokines in promoting COVID-19 severity [38], and explained the role of inflammatory cytokines in obesity [32,39,40], or even explored the network effect of adipokines and cytokines in SARS-CoV-2 [41], to our knowledge, there is no published evidence of a mediating role of inflammatory cytokines on the relationship between BMI and COVID-19 severity.

Other researchers have investigated the role of leptin on obesity [42,43] and leptin in COVID-19 patients [44,45]. Our study confirmed the statistically significant effect of high BMI levels on leptin and added to the evidence linking it to worsening COVID-19. The potential causal effect of BMI on leptin and leptin on COVID-19 severity were replicated and confirmed using different independent GWAS dataset. Despite its known pro-inflammatory effects, we were again unable to find any statistical evidence of a mediating role of leptin in the relationship between BMI and COVID-19 severity and to our knowledge no published study has reported this effect.

We also tested JAK-2 and lymphocyte percentage, both as causal consequence of BMI and as causes of COVID-19 severity. We found no evidence for either. Sun et al. studied the causal role of different white blood cells including lymphocytes percentage and found no significant role of lymphocytes in COVID-19 severity [46]. To our knowledge, no MR study has examined the role of JAK-2 with COVID-19 severity. However, JAK-2 was suggested as mediating the signalling pathway of inflammatory cytokines (i.e.: IL 6) [47] and anti-JAK-2 inhibitors are suggested for use to mitigate SARs-CoV-2 infection [48,49].

Our work has some limitations. The instruments were extracted from a GWAS study of 8,293 individuals which, although can be considered a large genetic study, is much smaller than current meta-analysis efforts covering over a million participants. This may affect the number of identified associated SNPs and limit our statistical power. In response, we lowered the p-value threshold we considered and used replication to verify our results. A major issue for all MR studies is pleiotropy. To ensure the validity of our results, we used the MR-Egger method to test for the presence of directional pleiotropy. The results indicated that our findings are unaffected by it, but pleiotropy remains a concern in all MR studies, more so when positive effects are reported. In two-sample MR approaches, it is important to use information from comparable populations as systematic changes between them may influence the results [50]. Here, our analysis is necessarily restricted to people of European ancestry, due to relevant GWAS data availability. It is unclear whether our conclusions hold for other ancestries, as some studies reported differences in the genetic architecture of the traits considered in this work among different ancestries, particularly in inflammatory cytokines secretion and function. Studying healthy individuals in GWAS might also not reflect the cytokine storm initiated in viral infection. GWAS, though powerful, has limitations for studying cytokine response to viruses as it captures genetic tendency, a snapshot, rather than the dynamic response during an infection. Finally, the participants of the population study were studied in general without specification to sex. Studies showed that inflammatory cytokines and COVID-19 severity are affected by hormonal status especially in females during menopause [51] and pregnancy [52].

Given the negative results of inflammatory cytokines mediation, other factors in addition to obesity affect COVID-19 severity. Patanavanich et al. found in a meta analysis that tobacco smoking is a progression risk factor for COVID-19 severity [53]. Others found type-2 diabetes as a metabolic risk factor that is linked to COVID-19 mortality in comparison to nondiabetic patients [54]. Coagulation dysfunction, elevated levels of fibrinogen, factor VIII, D-dimer and increased prothrombin time are associated with COVID-19 severity and mortality [55,56].

In our study, we used two-sample Mendelian randomization to study the mediating role of inflammatory cytokines and related traits in the link between COVID-19 severity and BMI. We investigated the implications of obesity for inflammatory cytokine levels and related traits, and their effect on COVID-19 severity. We found no secure evidence of proinflammatory cytokines, or related traits, mediating the relationship between BMI and increased risk of severe COVID-19 in European populations, despite estimating a potentially large mediating effect. Given the increasing levels of obesity worldwide and the establishment of COVID-19 as an endemic infection, the effectiveness of targeting cytokines to break the link between obesity and COVID-19 severity, remains an open question. Well-powered randomized control trials will be required to confirm any suitable cytokine related interventions to control the impact of obesity on COVID-19 as done previously with the WHO Solidarity study [57]. Our results in this respect remain inconclusive. Notably, our genetic and epidemiological findings do not negate the work of clinical and mechanistic evidence of elevated proinflammatory cytokines mainly IL 6, IL 1β, and TNF α to COVID-19 severity [58,59]. Other clinical and observational cohort studies have shown association of high cytokines levels in patients with respiratory failure and mortality [60]. Therefore, our results are considered as complementary and not replacing the clinical and observational studies. Following these findings, further work towards better understanding the genetics of the cytokine response, especially during the early stages of infections, and the genetic mechanisms of cytokine storm initiation will help us identify potential targets for intervention not only for response to COVID-19 but also for other similar infections and potentially future pandemic-inducing infections.

## Supplementary Material

supplementary tables Nov2025_zk.docx

## Data Availability

The data used is openly available.

## Abbreviations

BMI: Body mass index
2SMR: Two-sample Mendelian randomization
MVMR: Multivariable Mendelian randomization
JAK2: Janus Kinase 2
IVW: inverse variance weighted approach
