# Supplementary material for: Investigating the contribution of circulating inflammatory cytokines on the link between obesity and COVID-19
Source: Adipocyte. 2025 Dec 8;14(1):2596403. doi: 10.1080/21623945.2025.2596403 (PMC12688227; doi:10.1080/21623945.2025.2596403)
Supplement: supplementary tables Nov2025_zk.docx [file KADI_A_2596403_SM1065.docx]

**6 Supplementary data**

**Supplementary Table 1** Two sample Mendelian randomization analysis causal effect estimates of BMI (exposure) and inflammatory cytokines (outcome) using the inverse variance weighted analysis method. β:beta effect, SE: standard error~~.~~

| Exposure (BMI) | Outcome (cytokine) | | | β | SE | P-value | Egger intercept | Intercept p-value |
| --- | --- | --- | --- | --- | --- | --- | --- | --- |
| BMI | | **HGF** | 0.277 | | 0.070 | 8.00E-05 | -0.001 | 0.769 |
|  |  | **TRAIL** | 0.221 | | 0.068 | 1.09E-03 | -0.004 | 0.304 |
|  |  | **IL 13** | 0.226 | | 0.092 | 1.38E-02 | -0.003 | 0.548 |
|  |  | **IL 6** | 0.148 | | 0.064 | 2.01E-02 | 0.001 | 0.832 |
|  |  | **IL 7** | 0.196 | | 0.099 | 4.83E-02 | 0.001 | 0.816 |
|  |  | **CTACK** | -0.184 | | 0.097 | 5.60E-02 | -0.003 | 0.586 |
|  |  | **MCP 3** | 0.348 | | 0.183 | 5.73E-02 | -0.011 | 0.254 |
|  |  | **IL 9** | 0.180 | | 0.095 | 5.90E-02 | 0.002 | 0.710 |
|  |  | **IL 2** | 0.171 | | 0.093 | 6.44E-02 | -0.002 | 0.728 |
|  |  | **IL 1B** | 0.182 | | 0.100 | 6.86E-02 | 0.004 | 0.457 |
|  |  | **TNF A** | 0.166 | | 0.093 | 7.48E-02 | 0.001 | 0.842 |
|  |  | **IL 12 P70** | 0.118 | | 0.067 | 7.74E-02 | 0.002 | 0.654 |
|  |  | **MCP 1 MCAF** | 0.113 | | 0.064 | 7.79E-02 | -0.007 | 0.063 |
|  |  | **IL 1RA** | 0.162 | | 0.097 | 9.63E-02 | -0.002 | 0.684 |
|  |  | **IL 10** | 0.113 | | 0.069 | 1.04E-01 | -0.002 | 0.665 |
|  |  | **VEGF** | 0.104 | | 0.066 | 1.13E-01 | -0.005 | 0.120 |
|  |  | **IL 5** | 0.147 | | 0.096 | 1.24E-01 | -0.007 | 0.190 |
|  |  | **PDGF BB** | 0.094 | | 0.064 | 1.43E-01 | 0.000 | 0.902 |
|  |  | **IP 10** | 0.122 | | 0.090 | 1.74E-01 | -0.009 | 0.046 |
|  |  | **MIP 1A** | 0.129 | | 0.097 | 1.82E-01 | 0.003 | 0.539 |
|  |  | **B NGF** | 0.118 | | 0.093 | 2.04E-01 | -0.004 | 0.414 |
|  |  | **SCGF B** | 0.105 | | 0.091 | 2.49E-01 | 0.002 | 0.606 |
|  |  | **SDF 1A** | 0.070 | | 0.065 | 2.88E-01 | 0.002 | 0.525 |
|  |  | **MIF** | 0.100 | | 0.100 | 3.18E-01 | 0.005 | 0.331 |
|  |  | **FGF BASIC** | 0.054 | | 0.063 | 3.87E-01 | -0.002 | 0.499 |
|  |  | **MIP 1B** | 0.069 | | 0.081 | 3.98E-01 | -0.004 | 0.353 |
|  |  | **IL 18** | 0.075 | | 0.092 | 4.11E-01 | -0.007 | 0.124 |
|  |  | **IL 2RA** | 0.071 | | 0.090 | 4.30E-01 | -0.006 | 0.228 |
|  |  | **MIG** | -0.065 | | 0.090 | 4.67E-01 | -0.009 | 0.059 |
|  |  | **IL 8** | 0.064 | | 0.095 | 5.03E-01 | -0.010 | 0.048 |
|  |  | **G CSF** | 0.033 | | 0.065 | 6.11E-01 | 0.002 | 0.655 |
|  |  | **IL 16** | 0.048 | | 0.097 | 6.22E-01 | 0.003 | 0.635 |
|  |  | **IFN G** | 0.030 | | 0.063 | 6.31E-01 | -0.004 | 0.192 |
|  |  | **SCF** | 0.028 | | 0.064 | 6.58E-01 | -0.002 | 0.514 |
|  |  | **IL 4** | 0.028 | | 0.064 | 6.68E-01 | 0.000 | 0.993 |
|  |  | **TNF B** | 0.061 | | 0.148 | 6.80E-01 | 0.004 | 0.639 |
|  |  | **EOTAXIN** | 0.019 | | 0.064 | 7.68E-01 | 0.000 | 0.992 |
|  |  | **IL 17** | 0.017 | | 0.062 | 7.85E-01 | 0.000 | 0.899 |
|  |  | **M CSF** | 0.015 | | 0.116 | 8.95E-01 | 0.000 | 0.951 |
|  |  | **GROA** | -0.008 | | 0.092 | 9.34E-01 | -0.007 | 0.121 |
|  |  | **RANTES** | -0.002 | | 0.099 | 9.84E-01 | -0.004 | 0.443 |

**Supplementary Table 2** Two sample Mendelian randomization analysis causal effect estimates of inflammatory cytokines (exposure) to COVID-19 severity (outcome) using inverse variance weighted analysis method.

| Exposure  (Cytokines) | Outcome | OR | L95 | U95 | P-value | Egger intercept | Intercept p-value |
| --- | --- | --- | --- | --- | --- | --- | --- |
| TNF A | **COVID-19**  **severity** | 1.030 | 1.011 | 1.048 | 1.723E-03 | 0.578 | 0.456 |
| IL 8 |  | 1.017 | 1.000 | 1.034 | 4.876E-02 | 0.129 | 0.267 |
| IFN G |  | 1.084 | 0.985 | 1.183 | 1.113E-01 | 0.232 | 0.294 |
| SDF 1A |  | 0.979 | 0.952 | 1.007 | 1.325E-01 | 0.075 | 0.053 |
| IL 16 |  | 0.972 | 0.932 | 1.012 | 1.643E-01 | 0.424 | 0.631 |
| MIP 1B |  | 0.975 | 0.938 | 1.012 | 1.825E-01 | 0.340 | 0.278 |
| IL 1RA |  | 0.989 | 0.971 | 1.006 | 2.002E-01 | 0.456 | 0.262 |
| G CSF |  | 1.013 | 0.990 | 1.035 | 2.730E-01 | 0.202 | 0.599 |
| B NGF |  | 1.011 | 0.991 | 1.031 | 2.891E-01 | 0.896 | 0.077 |
| MCP 1 MCAF |  | 1.038 | 0.965 | 1.111 | 3.167E-01 | 0.413 | 0.216 |
| CTACK |  | 1.025 | 0.973 | 1.078 | 3.480E-01 | 0.425 | 0.446 |
| IL 18 |  | 0.968 | 0.895 | 1.040 | 3.770E-01 | 0.766 | 0.118 |
| PDGF BB |  | 1.024 | 0.968 | 1.080 | 4.061E-01 | 0.904 | 0.954 |
| FGF BASIC |  | 0.990 | 0.967 | 1.014 | 4.150E-01 | 0.665 | 0.728 |
| MCP 3 |  | 1.005 | 0.994 | 1.015 | 4.158E-01 | 0.317 | 0.562 |
| TNF B |  | 0.995 | 0.982 | 1.008 | 4.433E-01 | 0.720 | 0.680 |
| IP 10 |  | 1.034 | 0.947 | 1.121 | 4.546E-01 | 0.636 | 0.440 |
| VEGF |  | 1.012 | 0.974 | 1.049 | 5.410E-01 | 0.273 | 0.355 |
| TRAIL |  | 1.016 | 0.965 | 1.068 | 5.420E-01 | 0.118 | 0.038 |
| IL 4 |  | 0.965 | 0.849 | 1.081 | 5.492E-01 | 0.134 | 0.348 |
| M CSF |  | 0.996 | 0.981 | 1.010 | 5.606E-01 | 0.808 | 0.278 |
| MIP 1A |  | 0.995 | 0.977 | 1.012 | 5.667E-01 | 0.395 | 0.168 |
| IL 12 P70 |  | 1.014 | 0.965 | 1.062 | 5.804E-01 | 0.984 | 0.821 |
| IL 2 |  | 1.004 | 0.988 | 1.021 | 6.043E-01 | 0.832 | 0.483 |
| SCGF B |  | 0.987 | 0.934 | 1.040 | 6.289E-01 | 0.789 | 0.389 |
| IL 1B |  | 1.004 | 0.986 | 1.022 | 6.659E-01 | 0.938 | 0.079 |
| MIF |  | 0.980 | 0.874 | 1.087 | 7.154E-01 | 0.579 | 0.154 |
| IL 9 |  | 1.004 | 0.983 | 1.024 | 7.344E-01 | 0.276 | 0.010 |
| EOTAXIN |  | 1.003 | 0.936 | 1.071 | 9.219E-01 | 0.719 | 0.855 |
| GROA |  | 1.002 | 0.961 | 1.042 | 9.337E-01 | 0.833 | 0.303 |
| HGF |  | 0.892 | 0.800 | 0.984 | 1.458E-02 | 0.564 | 0.530 |
| MIG |  | 1.016 | 0.964 | 1.067 | 5.505E-01 | 0.301 | 0.345 |
| SCF |  | 0.902 | 0.659 | 1.145 | 4.041E-01 | 0.456 | 0.000 |
| IL 2RA |  | 1.004 | 0.987 | 1.020 | 6.849E-01 | 0.734 | 0.300 |
| IL 5 |  | 1.001 | 0.984 | 1.018 | 8.781E-01 | 0.737 | 0.315 |
| IL 6 |  | 0.975 | 0.950 | 1.000 | 4.523E-02 | 0.688 | 0.852 |
| IL 7 |  | 1.014 | 0.965 | 1.062 | 5.836E-01 | 0.729 | 0.885 |
| IL 10 |  | 0.989 | 0.923 | 1.055 | 7.416E-01 | 0.513 | 0.317 |
| IL 13 |  | 0.996 | 0.955 | 1.037 | 8.374E-01 | 0.607 | 0.968 |
| IL 17 |  | 1.007 | 0.906 | 1.107 | 8.952E-01 | 0.683 | 0.676 |
| RANTES |  | 1.003 | 0.939 | 1.067 | 9.216E-01 | 0.443 | 0.536 |

**Supplementary Table 3** Multivariable Mendelian randomization (MVMR) of BMI and inflammatory cytokines (exposures) with COVID-19 severity (outcome). SNPs: number of single nucleotide polymorphisms; OR odds ratio; L95, U95: conﬁdence interval. BNGF: B-lymphocyte-derived neurotrophic growth factor, MIP 1A: Macrophage Inflammatory Protein 1 Alpha, GROA: Growth-Regulated Oncogene Alpha, IL 2R2: Interleukin-2 Receptor Subunit Beta.

| Exposure (BMI+ cytokines) | Outcome  (COVID-19 severity) | | OR | L95 | | U95 | | P-value | |  |
| --- | --- | --- | --- | --- | --- | --- | --- | --- | --- | --- |
| BMI | | **COVID-19 (RELEASE 5)**  **ebi-a-GCST011073** | 0.968533 | | 0.812004 | | 1.125062 | | 6.89E-01 | |
| BNGF | |  | 1.025432 | | 0.985036 | | 1.065828 | | 2.23E-01 | |
| MIP 1A | |  | 0.972166 | | 0.920722 | | 1.02361 | | 2.82E-01 | |
| GROA | |  | 1.018828 | | 0.987188 | | 1.050468 | | 2.48E-01 | |
| IL 2RA | |  | 1.022909 | | 0.987461 | | 1.058356 | | 2.10E-01 | |

**Supplementary Table 4** :Causal Mediation Analysis of BMI mediation analysis with noncorrelated inflammatory cytokines. β (beta) causal effect – SE: standard error, CI-: lower confidence interval, CI+: higher confidence interval. Total effect estimated of BMI SNPs after MVMR. Direct effect: effect of BMI on COVID-19 with cytokines mediation. Indirect effect: total effect-direct effect.

|  | Effect % | β | SE | CI- | CI+ |
| --- | --- | --- | --- | --- | --- |
| Total effect | 100 | 0.196143 | 0.059616 | 0.079296 | 0.31299 |
| Direct effect | 47.92% | 0.094 | 0.053 | -0.00988 | 0.19788 |
| Indirect effect | 52.08% | 0.102143 | 0.079769 | -0.0542 | 0.258489 |

**Supplementary Table 5:** Summary of colocalization analysis of inflammatory cytokine IL_8. Marker: leading SNP; MIN.PVAL: min p_value, F_STAT: F_Statistics.

| marker | min.pval | F_stat | nsnps | H0 | H1 | H2 | H3 | H4 |
| --- | --- | --- | --- | --- | --- | --- | --- | --- |
| rs12075 | 9.97E-07 | 23.86426 | 2084 | 0.050542 | 0.892639 | 0.002952 | 0.052125 | 0.001741 |
| rs3786107 | 1.94E-06 | 22.69592 | 2795 | 0.094839 | 0.831886 | 0.005798 | 0.050795 | 0.016683 |
| rs141926526 | 1.96E-06 | 22.62064 | 782 | 0.184395 | 0.802919 | 0.002188 | 0.009515 | 0.000984 |
| rs12438669 | 2.60E-06 | 22.00057 | 538 | 0.005876 | 0.978738 | 8.72E-05 | 0.014505 | 0.000795 |
| rs2673604 | 3.29E-06 | 21.58224 | 1611 | 0.041999 | 0.925362 | 0.001364 | 0.030052 | 0.001222 |
| rs183628733 | 3.82E-06 | 21.3474 | 1385 | 0.02477 | 0.937687 | 0.000859 | 0.032502 | 0.004181 |
| rs116726256 | 4.26E-06 | 21.11487 | 489 | 0.00903 | 0.983593 | 6.17E-05 | 0.006704 | 0.000611 |
| rs75840288 | 4.85E-06 | 20.90145 | 585 | 0.094649 | 0.887631 | 0.00126 | 0.011734 | 0.004727 |
| rs17866606 | 5.12E-06 | 20.7936 | 1095 | 0.020705 | 0.879911 | 0.002235 | 0.094943 | 0.002207 |
| rs2552220 | 5.46E-06 | 20.60338 | 1798 | 0.027211 | 0.738414 | 0.00821 | 0.222779 | 0.003386 |
| rs77999880 | 5.55E-06 | 20.63804 | 284 | 0.077745 | 0.91518 | 0.00049 | 0.005733 | 0.000852 |
| rs79274420 | 6.75E-06 | 20.25349 | 784 | 0.109852 | 0.869634 | 0.002148 | 0.016989 | 0.001376 |
| rs1508652 | 9.62E-06 | 19.55107 | 1497 | 0.033168 | 0.936728 | 0.000966 | 0.02727 | 0.001869 |
| rs191947208 | 1.03E-05 | 19.45923 | 763 | 0.140126 | 0.845946 | 0.001804 | 0.010872 | 0.001252 |
| rs150154454 | 1.20E-05 | 19.18078 | 338 | 0.150227 | 0.842622 | 0.000893 | 0.004972 | 0.001286 |
| rs79371984 | 1.26E-05 | 19.0747 | 853 | 0.169047 | 0.819151 | 0.001848 | 0.008941 | 0.001014 |
| rs200034753 | 1.27E-05 | 19.05585 | 451 | 0.175569 | 0.815406 | 0.001441 | 0.00667 | 0.000914 |
| rs34600627 | 1.29E-05 | 19.05512 | 1699 | 0.107876 | 0.859127 | 0.003482 | 0.027717 | 0.001799 |
| rs12963102 | 1.36E-05 | 18.94409 | 609 | 0.06362 | 0.924839 | 0.00069 | 0.010021 | 0.000831 |
| rs12960757 | 1.36E-05 | 18.89305 | 2140 | 0.124959 | 0.809434 | 0.008094 | 0.052404 | 0.005109 |
| rs7132236 | 1.54E-05 | 18.67942 | 775 | 0.152913 | 0.831563 | 0.00218 | 0.011838 | 0.001506 |
| rs17255603 | 1.57E-05 | 18.63612 | 666 | 0.081188 | 0.882152 | 0.002763 | 0.029965 | 0.003931 |
| rs674363 | 1.57E-05 | 18.62227 | 1563 | 0.04644 | 0.926118 | 0.001238 | 0.024674 | 0.00153 |
| rs75294472 | 1.78E-05 | 18.40658 | 389 | 0.132198 | 0.856042 | 0.001472 | 0.009514 | 0.000774 |
| rs55972746 | 1.85E-05 | 18.41254 | 1916 | 0.071556 | 0.896625 | 0.00221 | 0.027684 | 0.001925 |
| rs2185618 | 2.00E-05 | 18.20147 | 1202 | 0.071374 | 0.90561 | 0.001448 | 0.018341 | 0.003228 |
| rs11128671 | 2.14E-05 | 18.03627 | 1865 | 0.078903 | 0.889376 | 0.002416 | 0.027227 | 0.002078 |
| rs2989733 | 2.16E-05 | 18.0625 | 277 | 0.042579 | 0.950192 | 0.000295 | 0.00656 | 0.000374 |
| rs116956220 | 2.28E-05 | 17.95104 | 492 | 0.100782 | 0.874833 | 0.002412 | 0.02092 | 0.001053 |
| rs17745654 | 2.30E-05 | 17.86843 | 1104 | 0.041763 | 0.940199 | 0.000731 | 0.016448 | 0.00086 |
| rs2651759 | 2.34E-05 | 17.9086 | 1209 | 0.083911 | 0.879787 | 0.002925 | 0.030644 | 0.002732 |
| rs10847827 | 2.46E-05 | 17.84899 | 1072 | 0.093117 | 0.888723 | 0.001619 | 0.015445 | 0.001096 |
| rs142252570 | 2.48E-05 | 17.79893 | 856 | 0.057648 | 0.925088 | 0.000954 | 0.0153 | 0.001009 |
| rs200196212 | 2.52E-05 | 17.74378 | 234 | 0.065112 | 0.927711 | 0.000396 | 0.005586 | 0.001195 |
| rs9819666 | 2.69E-05 | 17.6212 | 1096 | 0.113798 | 0.869694 | 0.001793 | 0.013691 | 0.001025 |
| rs61815292 | 2.78E-05 | 17.57219 | 870 | 0.018267 | 0.960547 | 0.000348 | 0.018276 | 0.002562 |
| rs55890755 | 2.80E-05 | 17.57169 | 1204 | 0.084814 | 0.886355 | 0.002371 | 0.024769 | 0.00169 |
| rs197766 | 2.83E-05 | 17.56645 | 719 | 0.095343 | 0.891935 | 0.001139 | 0.010643 | 0.000939 |
| rs17357714 | 2.88E-05 | 17.4636 | 1070 | 0.109922 | 0.855833 | 0.003678 | 0.028616 | 0.001952 |
| rs2292719 | 3.04E-05 | 17.36585 | 1360 | 0.056036 | 0.925363 | 0.001005 | 0.016581 | 0.001016 |
| rs74800745 | 3.21E-05 | 17.28421 | 846 | 0.083132 | 0.894301 | 0.001832 | 0.019699 | 0.001036 |
| rs56076760 | 3.26E-05 | 17.25059 | 719 | 0.160915 | 0.812558 | 0.004048 | 0.020411 | 0.002068 |
| rs144929914 | 3.34E-05 | 17.20605 | 483 | 0.107846 | 0.883528 | 0.000817 | 0.006673 | 0.001136 |
| rs4814847 | 3.41E-05 | 17.19999 | 920 | 0.092878 | 0.872024 | 0.002928 | 0.027435 | 0.004736 |
| rs9612022 | 3.42E-05 | 17.1065 | 1457 | 0.126591 | 0.839162 | 0.004235 | 0.028061 | 0.001951 |
| rs7289216 | 3.52E-05 | 17.09853 | 548 | 0.063888 | 0.927439 | 0.000531 | 0.007697 | 0.000444 |
| rs7705213 | 3.56E-05 | 17.09714 | 1747 | 0.147578 | 0.823011 | 0.004131 | 0.023025 | 0.002255 |
| rs12510130 | 3.62E-05 | 17.08001 | 833 | 0.087076 | 0.885896 | 0.002282 | 0.023195 | 0.001552 |
| rs114175475 | 3.69E-05 | 17.03795 | 1041 | 0.180282 | 0.76529 | 0.00984 | 0.041744 | 0.002844 |
| rs7872843 | 3.89E-05 | 16.89349 | 1514 | 0.055927 | 0.911154 | 0.001776 | 0.028927 | 0.002216 |
| rs9800493 | 3.98E-05 | 16.83453 | 1040 | 0.146965 | 0.831923 | 0.002999 | 0.016968 | 0.001145 |
| rs4715909 | 3.99E-05 | 16.83017 | 1238 | 0.04062 | 0.934773 | 0.000979 | 0.022517 | 0.001111 |
| rs62450817 | 4.06E-05 | 16.91597 | 2015 | 0.162071 | 0.791168 | 0.007356 | 0.035892 | 0.003513 |
| rs143319329 | 4.07E-05 | 16.85175 | 1078 | 0.166311 | 0.784681 | 0.007396 | 0.034832 | 0.00678 |
| rs142542588 | 4.08E-05 | 16.83146 | 149 | 0.187619 | 0.801331 | 0.001884 | 0.007965 | 0.001202 |
| rs710010 | 4.09E-05 | 16.82334 | 410 | 0.154076 | 0.825453 | 0.003033 | 0.016221 | 0.001217 |
| rs178582 | 4.13E-05 | 16.76654 | 91 | 0.050627 | 0.947749 | 7.67E-05 | 0.001423 | 0.000124 |
| rs115964966 | 4.18E-05 | 16.80135 | 194 | 0.141667 | 0.840485 | 0.002459 | 0.014543 | 0.000847 |
| rs79776685 | 4.18E-05 | 16.78079 | 117 | 0.146722 | 0.849684 | 0.000493 | 0.002831 | 0.000269 |
| rs9595579 | 4.19E-05 | 16.77059 | 626 | 0.089242 | 0.896208 | 0.001239 | 0.012427 | 0.000885 |
| rs117720701 | 4.25E-05 | 16.73728 | 638 | 0.184158 | 0.801173 | 0.002479 | 0.010762 | 0.001428 |
| rs9642725 | 4.29E-05 | 16.73248 | 1489 | 0.137333 | 0.838495 | 0.003188 | 0.019454 | 0.001529 |
| rs4806991 | 4.36E-05 | 16.6423 | 629 | 0.105355 | 0.877283 | 0.001752 | 0.014571 | 0.00104 |
| rs62042861 | 4.44E-05 | 16.66799 | 174 | 0.027702 | 0.969224 | 8.20E-05 | 0.002861 | 0.000132 |
| rs11925054 | 4.52E-05 | 16.64794 | 749 | 0.15536 | 0.826658 | 0.002505 | 0.013301 | 0.002176 |
| rs61781007 | 4.70E-05 | 16.566 | 363 | 0.066206 | 0.924884 | 0.000562 | 0.007833 | 0.000515 |
| rs72999615 | 4.73E-05 | 16.54287 | 1657 | 0.067713 | 0.900468 | 0.002072 | 0.027547 | 0.002199 |
| rs78807162 | 4.81E-05 | 16.52332 | 194 | 0.120329 | 0.865662 | 0.001597 | 0.011437 | 0.000976 |
| rs68102444 | 4.89E-05 | 16.51314 | 365 | 0.127055 | 0.867615 | 0.000643 | 0.004381 | 0.000307 |
| rs7143548 | 4.90E-05 | 16.53213 | 1762 | 0.073466 | 0.876988 | 0.003609 | 0.043065 | 0.002873 |
| rs55953468 | 5.04E-05 | 16.46616 | 1077 | 0.048192 | 0.926185 | 0.001198 | 0.023017 | 0.001408 |
| rs12551314 | 5.09E-05 | 16.45971 | 908 | 0.054768 | 0.854006 | 0.004457 | 0.069312 | 0.017456 |
| rs1051146 | 5.18E-05 | 16.42705 | 1147 | 0.06755 | 0.911895 | 0.00133 | 0.017938 | 0.001288 |
| rs7899518 | 5.32E-05 | 16.38786 | 1107 | 0.138089 | 0.729883 | 0.020216 | 0.10681 | 0.005002 |
| rs116929267 | 5.57E-05 | 16.25256 | 45 | 0.090655 | 0.907729 | 0.000133 | 0.001293 | 0.00019 |
| rs2138653 | 5.81E-05 | 16.14421 | 1172 | 0.119116 | 0.861587 | 0.002191 | 0.015835 | 0.001272 |
| rs186610413 | 6.07E-05 | 16.07734 | 93 | 0.171876 | 0.826085 | 0.000309 | 0.001459 | 0.000271 |
| rs34151221 | 6.15E-05 | 16.05665 | 784 | 0.137236 | 0.850942 | 0.001511 | 0.009359 | 0.000951 |
| rs2356868 | 6.28E-05 | 16 | 670 | 0.076552 | 0.903974 | 0.001422 | 0.016778 | 0.001274 |
| rs143251456 | 6.47E-05 | 15.97075 | 1353 | 0.080846 | 0.861233 | 0.003708 | 0.039393 | 0.01482 |
| rs55875103 | 6.65E-05 | 15.90818 | 858 | 0.134413 | 0.841409 | 0.003138 | 0.019626 | 0.001413 |
| rs56205209 | 6.74E-05 | 15.9297 | 487 | 0.076028 | 0.910726 | 0.000968 | 0.011585 | 0.000692 |
| rs140897629 | 6.79E-05 | 15.86879 | 288 | 0.122904 | 0.868264 | 0.000971 | 0.006824 | 0.001037 |
| rs71610430 | 6.84E-05 | 15.84096 | 687 | 0.156473 | 0.833022 | 0.001541 | 0.008191 | 0.000774 |
| rs34403890 | 7.06E-05 | 15.77678 | 1907 | 0.142822 | 0.809058 | 0.006832 | 0.038688 | 0.002599 |
| rs60585010 | 7.13E-05 | 15.74813 | 1491 | 0.112579 | 0.843393 | 0.004877 | 0.036522 | 0.002629 |
| rs189347896 | 7.16E-05 | 15.76394 | 177 | 0.175251 | 0.819344 | 0.00088 | 0.004091 | 0.000434 |
| rs79275043 | 7.16E-05 | 15.75277 | 375 | 0.184136 | 0.797043 | 0.003381 | 0.014611 | 0.00083 |
| rs200135360 | 7.18E-05 | 15.74131 | 158 | 0.055332 | 0.941236 | 0.000175 | 0.002965 | 0.000291 |
| rs2866308 | 7.23E-05 | 15.71779 | 1535 | 0.153825 | 0.80913 | 0.005562 | 0.02924 | 0.002242 |
| rs10535979 | 7.26E-05 | 15.72782 | 587 | 0.103612 | 0.887353 | 0.000874 | 0.007469 | 0.000692 |
| rs143987890 | 7.47E-05 | 15.69006 | 573 | 0.16112 | 0.830466 | 0.001216 | 0.006249 | 0.000949 |
| rs146283067 | 7.48E-05 | 15.68083 | 38 | 0.034863 | 0.964297 | 2.77E-05 | 0.000751 | 6.18E-05 |
| rs4987726 | 7.62E-05 | 15.64846 | 419 | 0.079611 | 0.912534 | 0.000579 | 0.00662 | 0.000656 |
| rs117207360 | 7.67E-05 | 15.62867 | 232 | 0.16562 | 0.827452 | 0.00109 | 0.00543 | 0.000408 |
| rs6583759 | 7.68E-05 | 15.65239 | 1635 | 0.137839 | 0.811141 | 0.006964 | 0.040963 | 0.003092 |
| rs71501109 | 7.70E-05 | 15.64518 | 552 | 0.166735 | 0.820522 | 0.001999 | 0.009819 | 0.000926 |
| rs9819803 | 7.81E-05 | 15.55711 | 1409 | 0.138403 | 0.834064 | 0.003693 | 0.022244 | 0.001596 |
| rs73591086 | 7.90E-05 | 15.58304 | 236 | 0.061852 | 0.926291 | 0.000573 | 0.008457 | 0.002827 |
| rs7403297 | 7.95E-05 | 15.51979 | 1578 | 0.090335 | 0.887402 | 0.001907 | 0.018721 | 0.001635 |
| rs77913145 | 7.98E-05 | 15.5656 | 307 | 0.22808 | 0.759159 | 0.00277 | 0.009193 | 0.000798 |
| rs112465461 | 8.03E-05 | 15.54336 | 169 | 0.152082 | 0.843755 | 0.00058 | 0.003195 | 0.000388 |
| rs9862992 | 8.06E-05 | 15.47111 | 293 | 0.17031 | 0.823925 | 0.000923 | 0.004453 | 0.000388 |
| rs7709723 | 8.27E-05 | 15.46275 | 632 | 0.081771 | 0.903947 | 0.001129 | 0.012473 | 0.00068 |
| rs140699642 | 8.28E-05 | 15.48578 | 154 | 0.143002 | 0.854102 | 0.000377 | 0.002231 | 0.000288 |
| rs1108344 | 8.30E-05 | 15.46581 | 727 | 0.171871 | 0.792614 | 0.006038 | 0.027824 | 0.001653 |
| rs6821962 | 8.42E-05 | 15.44575 | 1349 | 0.171414 | 0.795775 | 0.005464 | 0.025353 | 0.001994 |
| rs3908281 | 8.61E-05 | 15.41628 | 909 | 0.141268 | 0.843594 | 0.002049 | 0.012226 | 0.000864 |
| rs10507970 | 8.64E-05 | 15.43218 | 1641 | 0.088382 | 0.887633 | 0.002043 | 0.020509 | 0.001433 |
| rs12997351 | 8.77E-05 | 15.40601 | 1349 | 0.145123 | 0.831855 | 0.003216 | 0.018423 | 0.001383 |
| rs67198077 | 8.91E-05 | 15.3195 | 94 | 0.120692 | 0.877979 | 0.000151 | 0.001089 | 8.96E-05 |
| rs8134989 | 8.94E-05 | 15.3562 | 600 | 0.090461 | 0.887406 | 0.001971 | 0.019317 | 0.000844 |
| rs57603945 | 8.97E-05 | 15.32039 | 1121 | 0.096109 | 0.859117 | 0.004176 | 0.037297 | 0.003302 |
| rs75941693 | 9.05E-05 | 15.34405 | 344 | 0.123493 | 0.869716 | 0.000749 | 0.005252 | 0.000791 |
| rs2887150 | 9.50E-05 | 15.19454 | 832 | 0.078561 | 0.908129 | 0.00099 | 0.011431 | 0.00089 |
| rs2690021 | 9.61E-05 | 15.25779 | 507 | 0.13242 | 0.853483 | 0.001797 | 0.01157 | 0.00073 |
| rs75829172 | 9.64E-05 | 15.20748 | 518 | 0.161235 | 0.825031 | 0.002107 | 0.010767 | 0.000859 |
| rs218264 | 9.64E-05 | 15.20439 | 1344 | 0.138784 | 0.840291 | 0.0027 | 0.016334 | 0.001891 |
| rs17441830 | 9.67E-05 | 15.21505 | 816 | 0.153548 | 0.827264 | 0.002828 | 0.01522 | 0.00114 |
| rs7438943 | 9.68E-05 | 15.24585 | 1315 | 0.149684 | 0.822889 | 0.003917 | 0.021517 | 0.001993 |
| rs10242760 | 9.78E-05 | 15.21 | 583 | 0.113834 | 0.876748 | 0.00099 | 0.007615 | 0.000813 |
| rs72912365 | 9.83E-05 | 15.16787 | 474 | 0.13892 | 0.848743 | 0.001646 | 0.010044 | 0.000647 |
| rs10245886 | 9.83E-05 | 15.18925 | 1364 | 0.227647 | 0.739421 | 0.007104 | 0.023053 | 0.002775 |
| rs12554461 | 9.94E-05 | 15.12013 | 1948 | 0.154624 | 0.785702 | 0.00902 | 0.045808 | 0.004846 |
| rs35990266 | 9.95E-05 | 15.16449 | 631 | 0.105289 | 0.882282 | 0.001236 | 0.010348 | 0.000845 |
| rs62048844 | 0.000181 | 14.07041 | 157 | 0.105333 | 0.891368 | 0.000328 | 0.002763 | 0.000208 |
| rs111294808 | 0.000192 | 13.89776 | 135 | 0.104704 | 0.891087 | 0.000404 | 0.003407 | 0.000398 |
| rs71527181 | 0.000216 | 13.69477 | 142 | 0.176411 | 0.817628 | 0.000993 | 0.004576 | 0.000392 |
| rs55713548 | 0.00027 | 13.2496 | 294 | 0.209185 | 0.785761 | 0.000969 | 0.003625 | 0.000461 |
| rs12271665 | 0.000281 | 13.18898 | 322 | 0.206296 | 0.789482 | 0.000813 | 0.003101 | 0.000308 |
| rs10438423 | 0.000446 | 12.30792 | 201 | 0.217688 | 0.777282 | 0.001023 | 0.003634 | 0.000373 |
| rs111544221 | 0.000485 | 12.15758 | 64 | 0.174445 | 0.824278 | 0.000207 | 0.000962 | 0.000108 |
| rs62371864 | 0.000759 | 11.36454 | 959 | 0.205895 | 0.779258 | 0.002882 | 0.010896 | 0.001068 |
| rs112560015 | 0.000914 | 10.98909 | 52 | 0.224057 | 0.774951 | 0.000205 | 0.000692 | 9.51E-05 |
| rs6442401 | 0.000931 | 11.00112 | 317 | 0.212374 | 0.777302 | 0.002065 | 0.007534 | 0.000725 |
| rs148116035 | 0.000964 | 10.89382 | 164 | 0.23566 | 0.754011 | 0.002248 | 0.007137 | 0.000943 |
| rs74714351 | 0.001219 | 10.45903 | 392 | 0.1913 | 0.800973 | 0.001378 | 0.005754 | 0.000596 |
| rs17119359 | 0.001258 | 10.41478 | 179 | 0.207042 | 0.753976 | 0.008163 | 0.029663 | 0.001156 |
| rs650052 | 0.00133 | 10.31981 | 124 | 0.23781 | 0.759334 | 0.000632 | 0.001998 | 0.000226 |
| rs62346386 | 0.001531 | 10.03175 | 128 | 0.15959 | 0.836685 | 0.000564 | 0.00294 | 0.000221 |
| rs201356276 | 0.001653 | 9.896103 | 129 | 0.252144 | 0.744168 | 0.000853 | 0.002492 | 0.000343 |
| rs12228620 | 0.001656 | 9.888849 | 92 | 0.16931 | 0.828611 | 0.000321 | 0.001546 | 0.000212 |
| rs3019665 | 0.001679 | 9.845759 | 261 | 0.216575 | 0.776929 | 0.001339 | 0.004789 | 0.000368 |
| rs138053740 | 0.002136 | 9.418889 | 168 | 0.221248 | 0.77497 | 0.000778 | 0.002707 | 0.000297 |
| rs1069381 | 0.00239 | 9.233 | 87 | 0.182797 | 0.813478 | 0.000629 | 0.002761 | 0.000335 |
| rs62154883 | 0.002612 | 9.065791 | 78 | 0.179273 | 0.818761 | 0.000328 | 0.001476 | 0.000162 |
| rs10174067 | 0.002999 | 8.802421 | 11 | 0.262766 | 0.736692 | 0.000135 | 0.000352 | 5.47E-05 |
| rs148272384 | 0.007266 | 7.198753 | 12 | 0.335365 | 0.663823 | 0.000258 | 0.00047 | 8.38E-05 |
| rs180707113 | 0.009406 | 6.744863 | 15 | 0.398943 | 0.600191 | 0.000332 | 0.000463 | 7.17E-05 |

**Supplementary Table 6:** Summary of colocalization analysis of inflammatory cytokine TNF α. : leading SNP; MIN.PVAL: min p_value, F_STAT: F_Statistics.

| MARKER | MIN.PVAL | F_STAT | NSNPS | H0 | H1 | H2 | H3 | H4 |
| --- | --- | --- | --- | --- | --- | --- | --- | --- |
| rs111332265 | 7.91E-07 | 24.37310752 | 2599 | 0.02698 | 0.933828 | 0.001029 | 0.035618 | 0.002544 |
| rs150978375 | 1.11E-06 | 23.72442316 | 675 | 0.129694 | 0.786734 | 0.011223 | 0.068015 | 0.004335 |
| rs10834997 | 1.53E-06 | 23.08502197 | 2033 | 0.011049 | 0.956927 | 0.000348 | 0.030093 | 0.001584 |
| rs149420276 | 2.15E-06 | 22.43654633 | 382 | 0.034976 | 0.959567 | 0.000175 | 0.00478 | 0.000502 |
| rs79105320 | 2.21E-06 | 22.41946064 | 602 | 0.129745 | 0.856361 | 0.001555 | 0.010227 | 0.002113 |
| rs72725197 | 3.55E-06 | 21.48581462 | 359 | 0.068352 | 0.924743 | 0.000425 | 0.005729 | 0.000751 |
| rs8121916 | 5.19E-06 | 20.75674126 | 1671 | 0.021833 | 0.943509 | 0.000747 | 0.032286 | 0.001625 |
| rs12351466 | 1.04E-05 | 19.40759678 | 1623 | 0.100315 | 0.835967 | 0.006353 | 0.052919 | 0.004446 |
| rs72841564 | 1.25E-05 | 19.05774078 | 562 | 0.096763 | 0.8833 | 0.00185 | 0.016869 | 0.001218 |
| rs2292719 | 1.32E-05 | 18.93425858 | 1898 | 0.044116 | 0.928884 | 0.001151 | 0.024235 | 0.001614 |
| rs149323174 | 1.36E-05 | 18.90019888 | 734 | 0.141128 | 0.840766 | 0.002412 | 0.014349 | 0.001346 |
| rs7557984 | 1.37E-05 | 18.96462019 | 1513 | 0.092736 | 0.876025 | 0.002815 | 0.026579 | 0.001845 |
| rs235379 | 1.52E-05 | 18.6665023 | 159 | 0.06462 | 0.932996 | 0.000142 | 0.00204 | 0.000201 |
| rs12128908 | 1.61E-05 | 18.60711469 | 154 | 0.026573 | 0.971082 | 5.62E-05 | 0.002038 | 0.000251 |
| rs12696114 | 1.65E-05 | 18.6082276 | 2165 | 0.108439 | 0.841637 | 0.005256 | 0.040778 | 0.003889 |
| rs71547119 | 1.67E-05 | 18.55317314 | 466 | 0.121023 | 0.866207 | 0.001475 | 0.010544 | 0.000751 |
| rs3750434 | 1.71E-05 | 18.55071366 | 775 | 0.05498 | 0.931761 | 0.000704 | 0.011928 | 0.000627 |
| rs10424794 | 1.75E-05 | 18.44148348 | 231 | 0.06119 | 0.934625 | 0.00024 | 0.003658 | 0.000287 |
| rs117402431 | 1.77E-05 | 18.38896194 | 384 | 0.108158 | 0.880947 | 0.0011 | 0.008936 | 0.000859 |
| rs150603730 | 1.82E-05 | 18.36734694 | 136 | 0.130077 | 0.8663 | 0.000417 | 0.002742 | 0.000464 |
| rs186202801 | 1.89E-05 | 18.3072922 | 246 | 0.157464 | 0.838543 | 0.000574 | 0.003042 | 0.000378 |
| rs72766753 | 2.11E-05 | 18.08907227 | 945 | 0.094094 | 0.887441 | 0.001648 | 0.015533 | 0.001284 |
| rs78306826 | 2.13E-05 | 18.02632591 | 994 | 0.035523 | 0.930352 | 0.001203 | 0.031487 | 0.001436 |
| rs13059921 | 2.34E-05 | 17.943696 | 426 | 0.027859 | 0.62418 | 0.014752 | 0.330446 | 0.002763 |
| rs1899347 | 2.58E-05 | 17.71200704 | 552 | 0.138594 | 0.850616 | 0.001396 | 0.008551 | 0.000843 |
| rs116264291 | 2.58E-05 | 17.71205403 | 86 | 0.083501 | 0.914911 | 0.000121 | 0.001312 | 0.000156 |
| rs4689050 | 2.61E-05 | 17.66889434 | 1626 | 0.117787 | 0.837503 | 0.004468 | 0.031719 | 0.008523 |
| rs61735793 | 2.63E-05 | 17.66798655 | 154 | 0.161539 | 0.835201 | 0.000481 | 0.002464 | 0.000315 |
| rs17232800 | 2.86E-05 | 17.48919399 | 662 | 0.079008 | 0.909286 | 0.000844 | 0.009701 | 0.001161 |
| rs17319264 | 2.91E-05 | 17.47449662 | 361 | 0.165192 | 0.825213 | 0.001481 | 0.007379 | 0.000734 |
| rs80278206 | 2.92E-05 | 17.45083247 | 786 | 0.065787 | 0.91965 | 0.00091 | 0.012712 | 0.000941 |
| rs12407607 | 2.96E-05 | 17.42155009 | 1583 | 0.069488 | 0.389072 | 0.078778 | 0.440951 | 0.021711 |
| rs79975430 | 3.01E-05 | 17.42822566 | 640 | 0.174692 | 0.804078 | 0.003552 | 0.016328 | 0.00135 |
| rs141112349 | 3.01E-05 | 17.40641079 | 601 | 0.256229 | 0.73387 | 0.002357 | 0.006736 | 0.000809 |
| rs144753551 | 3.04E-05 | 17.35534857 | 258 | 0.094827 | 0.898135 | 0.000633 | 0.005982 | 0.000423 |
| rs4840094 | 3.05E-05 | 17.35534857 | 1821 | 0.092723 | 0.878878 | 0.002532 | 0.023994 | 0.001873 |
| rs28542845 | 3.16E-05 | 17.3288264 | 1114 | 0.078592 | 0.897938 | 0.00176 | 0.020091 | 0.001619 |
| rs8093341 | 3.18E-05 | 17.36678051 | 769 | 0.113679 | 0.873839 | 0.001329 | 0.010204 | 0.000948 |
| rs71070949 | 3.19E-05 | 17.35649717 | 1219 | 0.172923 | 0.794808 | 0.005339 | 0.02452 | 0.002409 |
| rs79884832 | 3.23E-05 | 17.2652557 | 632 | 0.033778 | 0.954213 | 0.000386 | 0.0109 | 0.000722 |
| rs11564228 | 3.53E-05 | 17.07704068 | 496 | 0.155425 | 0.83639 | 0.001189 | 0.006386 | 0.00061 |
| rs78016169 | 3.69E-05 | 17.04182159 | 616 | 0.135994 | 0.841821 | 0.002881 | 0.01781 | 0.001494 |
| rs7199531 | 3.71E-05 | 17.01087303 | 1223 | 0.111963 | 0.861138 | 0.002914 | 0.022401 | 0.001584 |
| rs74798810 | 3.74E-05 | 17.00787212 | 428 | 0.072044 | 0.917571 | 0.000699 | 0.008878 | 0.000809 |
| rs12884079 | 3.75E-05 | 17.05757155 | 1469 | 0.104901 | 0.853888 | 0.004241 | 0.034506 | 0.002464 |
| rs4779639 | 3.75E-05 | 16.941456 | 695 | 0.105134 | 0.882604 | 0.00122 | 0.01023 | 0.000812 |
| rs138802229 | 3.80E-05 | 16.97014881 | 187 | 0.100692 | 0.892078 | 0.000702 | 0.006202 | 0.000326 |
| rs17746790 | 3.91E-05 | 16.92675024 | 893 | 0.152458 | 0.828839 | 0.002713 | 0.014734 | 0.001255 |
| rs12133469 | 4.03E-05 | 16.87517472 | 389 | 0.039287 | 0.954945 | 0.000211 | 0.00512 | 0.000438 |
| rs67921043 | 4.19E-05 | 16.77576683 | 320 | 0.098888 | 0.897259 | 0.000357 | 0.003229 | 0.000268 |
| rs13201977 | 4.20E-05 | 16.78306244 | 607 | 0.148282 | 0.831687 | 0.00269 | 0.015053 | 0.002288 |
| rs201693167 | 4.22E-05 | 16.75005354 | 887 | 0.179367 | 0.807462 | 0.00218 | 0.0098 | 0.001191 |
| rs74162546 | 4.26E-05 | 16.73931304 | 651 | 0.164133 | 0.822703 | 0.001966 | 0.009835 | 0.001363 |
| rs2760482 | 4.42E-05 | 16.62642232 | 897 | 0.071551 | 0.869831 | 0.004325 | 0.052555 | 0.001739 |
| rs117509142 | 4.48E-05 | 16.65947794 | 673 | 0.198535 | 0.787868 | 0.002518 | 0.009976 | 0.001103 |
| rs57570563 | 4.54E-05 | 16.67768654 | 1223 | 0.115518 | 0.852171 | 0.003606 | 0.026584 | 0.002122 |
| rs56065840 | 4.57E-05 | 16.61113222 | 326 | 0.173605 | 0.820039 | 0.001022 | 0.00481 | 0.000524 |
| rs3188410 | 4.59E-05 | 16.64221564 | 1532 | 0.161228 | 0.78591 | 0.008539 | 0.041604 | 0.002719 |
| rs6882830 | 4.60E-05 | 16.58353898 | 467 | 0.030277 | 0.956173 | 0.000403 | 0.012729 | 0.000417 |
| rs9421143 | 4.73E-05 | 16.56259464 | 1483 | 0.177722 | 0.779584 | 0.007341 | 0.032182 | 0.003171 |
| rs9648731 | 4.88E-05 | 16.44753087 | 1261 | 0.181307 | 0.798592 | 0.003443 | 0.015155 | 0.001502 |
| rs1885929 | 4.91E-05 | 16.44428917 | 746 | 0.133634 | 0.846123 | 0.002575 | 0.016287 | 0.001381 |
| rs76759473 | 4.97E-05 | 16.48402402 | 1460 | 0.151587 | 0.826958 | 0.003076 | 0.016772 | 0.001606 |
| rs7292316 | 5.04E-05 | 16.45549326 | 657 | 0.162472 | 0.807973 | 0.00443 | 0.021983 | 0.003142 |
| rs149284461 | 5.06E-05 | 16.4214307 | 188 | 0.174486 | 0.820587 | 0.000803 | 0.003758 | 0.000366 |
| rs7899518 | 5.14E-05 | 16.44753087 | 765 | 0.17164 | 0.778867 | 0.008487 | 0.038478 | 0.002528 |
| rs2883445 | 5.15E-05 | 16.38119114 | 1975 | 0.179971 | 0.786292 | 0.005859 | 0.025586 | 0.002292 |
| rs118148307 | 5.16E-05 | 16.41624278 | 449 | 0.15448 | 0.836717 | 0.001262 | 0.00682 | 0.00072 |
| rs61692633 | 5.21E-05 | 16.38420587 | 1156 | 0.125445 | 0.842296 | 0.003726 | 0.024987 | 0.003546 |
| rs114402660 | 5.30E-05 | 16.33871736 | 286 | 0.212701 | 0.779732 | 0.001515 | 0.005534 | 0.000518 |
| rs11264870 | 5.45E-05 | 16.28292998 | 1494 | 0.209868 | 0.750214 | 0.008033 | 0.028696 | 0.003189 |
| rs56332338 | 5.53E-05 | 16.29890377 | 1591 | 0.134465 | 0.827351 | 0.004981 | 0.03063 | 0.002573 |
| rs1727422 | 5.56E-05 | 16.25196463 | 2025 | 1.93E-05 | 0.000106 | 0.140786 | 0.770952 | 0.088138 |
| rs75056204 | 5.58E-05 | 16.24413816 | 261 | 0.087691 | 0.895144 | 0.001443 | 0.014695 | 0.001026 |
| rs719203 | 5.63E-05 | 16.19028574 | 745 | 0.109955 | 0.878123 | 0.001236 | 0.009856 | 0.00083 |
| rs11353815 | 5.65E-05 | 16.22470201 | 1828 | 0.136721 | 0.831413 | 0.003978 | 0.024169 | 0.003718 |
| rs11124033 | 5.68E-05 | 16.15786781 | 898 | 0.163869 | 0.809801 | 0.004178 | 0.020629 | 0.001523 |
| rs3138092 | 5.81E-05 | 16.14578512 | 731 | 0.054287 | 0.935739 | 0.000502 | 0.008635 | 0.000837 |
| rs6979813 | 5.90E-05 | 16.19413371 | 770 | 0.172692 | 0.812706 | 0.002391 | 0.011238 | 0.000973 |
| rs2204283 | 6.28E-05 | 15.97194214 | 992 | 0.090497 | 0.867207 | 0.003789 | 0.036283 | 0.002225 |
| rs59557443 | 6.35E-05 | 15.99532745 | 365 | 0.203548 | 0.790139 | 0.001194 | 0.00462 | 0.0005 |
| rs116418527 | 6.43E-05 | 16.02997657 | 933 | 0.109581 | 0.874683 | 0.001636 | 0.013051 | 0.001049 |
| rs117234146 | 6.74E-05 | 15.9076481 | 612 | 0.078332 | 0.789092 | 0.011649 | 0.11729 | 0.003636 |
| rs17558821 | 6.94E-05 | 15.83781149 | 262 | 0.072146 | 0.918075 | 0.000671 | 0.008518 | 0.00059 |
| rs56160232 | 6.97E-05 | 15.78608567 | 443 | 0.092952 | 0.897765 | 0.000824 | 0.007944 | 0.000515 |
| rs12681460 | 7.02E-05 | 15.79147883 | 1841 | 0.169272 | 0.789372 | 0.006046 | 0.028155 | 0.007155 |
| rs117872968 | 7.08E-05 | 15.77428946 | 533 | 0.135518 | 0.848819 | 0.00203 | 0.0127 | 0.000933 |
| rs61113779 | 7.15E-05 | 15.75716116 | 956 | 0.158 | 0.822004 | 0.003024 | 0.015721 | 0.001251 |
| rs78353284 | 7.30E-05 | 15.7232912 | 295 | 0.168121 | 0.822149 | 0.001517 | 0.00739 | 0.000824 |
| rs72652719 | 7.33E-05 | 15.72338882 | 226 | 0.123254 | 0.871667 | 0.00059 | 0.004157 | 0.000332 |
| rs9876613 | 7.43E-05 | 15.68369809 | 685 | 0.087046 | 0.898804 | 0.001146 | 0.011817 | 0.001187 |
| rs3748511 | 7.49E-05 | 15.63842976 | 548 | 0.175071 | 0.803355 | 0.003596 | 0.016475 | 0.001503 |
| rs6142718 | 7.73E-05 | 15.66058714 | 344 | 0.021816 | 0.971247 | 0.000135 | 0.006007 | 0.000795 |
| rs4659108 | 7.80E-05 | 15.61333729 | 492 | 0.174745 | 0.818868 | 0.001038 | 0.004854 | 0.000495 |
| rs6707510 | 7.94E-05 | 15.62511762 | 303 | 0.158184 | 0.835858 | 0.000888 | 0.004678 | 0.000393 |
| rs192144305 | 8.09E-05 | 15.54552283 | 232 | 0.206845 | 0.784602 | 0.001638 | 0.006182 | 0.000732 |
| rs28491355 | 8.11E-05 | 15.53185013 | 738 | 0.191269 | 0.798042 | 0.00187 | 0.007787 | 0.001033 |
| rs9969435 | 8.28E-05 | 15.49086637 | 861 | 0.12055 | 0.83449 | 0.005236 | 0.036206 | 0.003518 |
| rs10838433 | 8.29E-05 | 15.45919422 | 1441 | 0.156312 | 0.807619 | 0.005484 | 0.028318 | 0.002267 |
| rs118162834 | 8.39E-05 | 15.44534575 | 511 | 0.162572 | 0.811686 | 0.003902 | 0.019434 | 0.002406 |
| rs7358464 | 8.54E-05 | 15.43159954 | 379 | 0.132801 | 0.860221 | 0.000858 | 0.005543 | 0.000577 |
| rs115330808 | 8.62E-05 | 15.41337268 | 410 | 0.186935 | 0.805585 | 0.001113 | 0.004757 | 0.00161 |
| rs181890788 | 8.71E-05 | 15.38910799 | 141 | 0.152887 | 0.84322 | 0.000564 | 0.003091 | 0.000238 |
| rs3785273 | 8.99E-05 | 15.31114976 | 1115 | 0.140339 | 0.838393 | 0.00282 | 0.01683 | 0.001619 |
| rs2734697 | 9.00E-05 | 15.37316909 | 1139 | 0.12766 | 0.849363 | 0.002786 | 0.018524 | 0.001667 |
| rs11692196 | 9.05E-05 | 15.29368623 | 216 | 0.153366 | 0.841973 | 0.000655 | 0.003575 | 0.000431 |
| rs150849866 | 9.34E-05 | 15.25898804 | 329 | 0.292116 | 0.702761 | 0.001386 | 0.003323 | 0.000414 |
| rs10765577 | 9.37E-05 | 15.25478702 | 1369 | 0.145127 | 0.79245 | 0.009092 | 0.04962 | 0.003712 |
| rs114437437 | 9.45E-05 | 15.23501342 | 214 | 0.141831 | 0.853001 | 0.000695 | 0.004167 | 0.000305 |
| rs11930273 | 9.59E-05 | 15.1934727 | 737 | 0.136103 | 0.848496 | 0.001975 | 0.012295 | 0.001132 |
| rs12133591 | 9.61E-05 | 15.15203879 | 197 | 0.02059 | 0.974144 | 0.000103 | 0.004878 | 0.000286 |
| rs28473246 | 9.75E-05 | 15.14848338 | 722 | 0.146697 | 0.840642 | 0.001739 | 0.00995 | 0.000972 |
| rs7107378 | 9.94E-05 | 15.16090582 | 734 | 0.12032 | 0.868333 | 0.001279 | 0.009221 | 0.000846 |
| rs78569615 | 0.000107 | 15.00960852 | 690 | 0.177161 | 0.811449 | 0.001886 | 0.008628 | 0.000876 |
| rs137984817 | 0.000108 | 15.00025195 | 190 | 0.205156 | 0.78807 | 0.001295 | 0.004946 | 0.000533 |
| rs28666561 | 0.000108 | 14.98223841 | 674 | 0.160647 | 0.828372 | 0.00165 | 0.008497 | 0.000833 |
| rs12913535 | 0.000114 | 14.89562275 | 352 | 0.153261 | 0.839699 | 0.000994 | 0.005431 | 0.000615 |
| rs114482869 | 0.000114 | 14.89710978 | 117 | 0.148617 | 0.849039 | 0.000328 | 0.001863 | 0.000152 |
| rs13332133 | 0.000114 | 14.87909237 | 1412 | 0.089266 | 0.878542 | 0.002507 | 0.024635 | 0.00505 |
| rs145291982 | 0.000121 | 14.78434277 | 509 | 0.052868 | 0.933595 | 0.000681 | 0.012009 | 0.000847 |
| rs10435712 | 0.000121 | 14.79603035 | 149 | 0.070726 | 0.927097 | 0.000146 | 0.001911 | 0.00012 |
| rs111545888 | 0.000127 | 14.67948598 | 323 | 0.151398 | 0.842592 | 0.000845 | 0.004685 | 0.000481 |
| rs1035539 | 0.00013 | 14.62432073 | 1023 | 0.189142 | 0.793992 | 0.003037 | 0.012739 | 0.001089 |
| rs58955905 | 0.000141 | 14.51776866 | 114 | 0.124728 | 0.873324 | 0.000226 | 0.001572 | 0.000149 |
| rs111879377 | 0.000155 | 14.29693202 | 128 | 0.158897 | 0.838344 | 0.00041 | 0.002148 | 0.000201 |
| rs11636217 | 0.000183 | 14.00063126 | 182 | 0.151856 | 0.84461 | 0.000502 | 0.00278 | 0.000252 |
| rs148066616 | 0.000235 | 13.52270207 | 102 | 0.146178 | 0.851297 | 0.000349 | 0.002017 | 0.000159 |
| rs62016144 | 0.000236 | 13.53580976 | 326 | 0.124346 | 0.837337 | 0.004751 | 0.031943 | 0.001624 |
| rs73610561 | 0.000237 | 13.53572076 | 499 | 0.151157 | 0.839309 | 0.001355 | 0.007512 | 0.000667 |
| rs3095917 | 0.000296 | 13.12696649 | 171 | 0.107196 | 0.887078 | 0.00057 | 0.004693 | 0.000463 |
| rs6845128 | 0.00034 | 12.8499122 | 243 | 0.216616 | 0.779937 | 0.000688 | 0.002467 | 0.000292 |
| rs140842863 | 0.000373 | 12.67147472 | 208 | 0.21177 | 0.782998 | 0.000947 | 0.003461 | 0.000825 |
| rs1125280 | 0.000495 | 12.12157582 | 221 | 0.169197 | 0.827006 | 0.000598 | 0.002908 | 0.000291 |
| rs191892074 | 0.000672 | 11.56856659 | 137 | 0.281673 | 0.712653 | 0.001479 | 0.003707 | 0.000488 |
| rs35923610 | 0.000686 | 11.51059835 | 178 | 0.215743 | 0.779326 | 0.001008 | 0.003624 | 0.0003 |
| rs56237316 | 0.000757 | 11.33988984 | 47 | 0.218062 | 0.780768 | 0.000239 | 0.000836 | 9.44E-05 |
| rs62042556 | 0.000847 | 11.12878988 | 68 | 0.285592 | 0.713064 | 0.000362 | 0.00089 | 9.11E-05 |
| rs8043242 | 0.000892 | 11.02793403 | 740 | 0.210373 | 0.771056 | 0.003693 | 0.013516 | 0.001363 |
| rs74379625 | 0.000962 | 10.89 | 81 | 0.232306 | 0.766232 | 0.000317 | 0.001032 | 0.000113 |
| rs10024656 | 0.000977 | 10.85421549 | 239 | 0.219848 | 0.77631 | 0.00079 | 0.002779 | 0.000272 |
| rs1363592 | 0.000986 | 10.86427054 | 178 | 0.234064 | 0.763291 | 0.000555 | 0.001794 | 0.000295 |
| rs60169937 | 0.001038 | 10.76722872 | 324 | 0.189475 | 0.803776 | 0.001162 | 0.00491 | 0.000677 |
| rs141467296 | 0.001071 | 10.69371898 | 55 | 0.175494 | 0.822522 | 0.000303 | 0.001361 | 0.00032 |
| rs117234384 | 0.001089 | 10.66157553 | 90 | 0.207893 | 0.788966 | 0.000607 | 0.002276 | 0.000258 |
| rs13435460 | 0.001248 | 10.41634845 | 44 | 0.239292 | 0.759543 | 0.00026 | 0.000801 | 0.000104 |
| rs76115252 | 0.001586 | 9.964377239 | 141 | 0.11424 | 0.8807 | 0.000526 | 0.004015 | 0.00052 |
| rs116253719 | 0.00215 | 9.420177513 | 144 | 0.24627 | 0.751101 | 0.000605 | 0.001833 | 0.000191 |
| rs55873401 | 0.002564 | 9.098900356 | 77 | 0.182817 | 0.815478 | 0.000286 | 0.001255 | 0.000164 |
| rs6733784 | 0.004254 | 8.187363492 | 53 | 0.293119 | 0.705944 | 0.000257 | 0.000606 | 7.43E-05 |
| rs6685637 | 0.006182 | 7.473882808 | 95 | 0.309936 | 0.688705 | 0.000395 | 0.000868 | 9.54E-05 |
